# The Alzheimer’s disease drug development landscape

**DOI:** 10.1186/s13195-021-00927-z

**Published:** 2021-11-11

**Authors:** Pieter van Bokhoven, Arno de Wilde, Lisa Vermunt, Prisca S. Leferink, Sasja Heetveld, Jeffrey Cummings, Philip Scheltens, Everard G. B. Vijverberg

**Affiliations:** 1 Industry Alliance Office, Amsterdam Neuroscience, Amsterdam University Medical Centers, Amsterdam, The Netherlands; 2 Life Science Partners (LSP), Amsterdam, The Netherlands; 3 Alzheimer Center Amsterdam, Department of Neurology, Amsterdam Neuroscience, Vrije Universiteit Amsterdam, Amsterdam UMC, Amsterdam, The Netherlands; 4 Neurochemistry Laboratory, Department of Clinical Chemistry, Amsterdam Neuroscience, Vrije Universiteit Amsterdam, Amsterdam UMC, Amsterdam, The Netherlands; 5 Chambers-Grundy Center for Transformative Neuroscience, Department of Brain Health, School of Integrated Health Sciences, University of Nevada Las Vegas (UNLV), Las Vegas, NV USA

**Keywords:** Alzheimer’s disease, Drug development, Drug targets, Therapy

## Abstract

**Background:**

Alzheimer’s disease (AD) is a devastating neurodegenerative disease leading to dementia. The field has made significant progress over the last 15 years. AD diagnosis has shifted from syndromal, based on signs and symptoms, to a biomarker construct based on the pathological hallmarks of the disease: amyloid β deposition, pathologic tau, and neurodegeneration. Numerous genetic risk factors for sporadic AD have been identified, providing further insight into the molecular underpinnings of the disease. For the last two decades, however, drug development for AD has been proven to be particularly challenging. Here, we provide a unique overview of the drug development landscape for AD. By comparing preclinical and clinical drug development pipelines, we aim to describe trends and differences regarding target classes and therapeutic modalities in preclinical and clinical development.

**Methods:**

We analyzed proprietary and public databases and company websites for drugs in preclinical development for AD by the pharmaceutical industry and major clinical trial registries for drugs in clinical development for AD. Drugs were categorized by target class and treatment modality.

**Results:**

We found a higher proportion of preclinical interventions targeting molecular pathways associated with sporadic AD genetic risk variants, compared to clinical stage interventions. These include apolipoprotein E (ApoE) and lipids, lysosomal/endosomal targets, and proteostasis. Further, we observed a trend suggesting that more traditional therapeutic modalities are developed for these novel targets, while more novel treatment modalities such as gene therapies and enzyme treatments are in development for more traditional targets such as amyloid β and tau. Interestingly, the percentage of amyloid β targeting therapies in preclinical development (19.2%) is even higher than the percentage in clinical development (10.7%), indicating that diversification away from interventions targeting amyloid-beta has not materialized. Inflammation is the second most popular target class in both preclinical and clinical development.

**Conclusions:**

Our observations show that the AD drug development pipeline is diversifying in terms of targets and treatment modalities, while amyloid-targeting therapies remain a prominent avenue of development as well. To further advance AD drug development, novel companion diagnostics are needed that are directed at disease mechanisms related to genetic risk factors of AD, both for patient stratification and assessment of therapeutic efficacy in clinical trials.

**Supplementary Information:**

The online version contains supplementary material available at 10.1186/s13195-021-00927-z.

## Background

Approximately 47 million people worldwide are living with dementia of which most are affected with a devastating form of a neurodegenerative disorder called Alzheimer’s disease [1]. Clinically, AD shows symptoms of progressive cognitive impairment ultimately leading to dementia. Patients with AD and their caregivers are in urgent need of therapeutic interventions that prevent, halt, slow, or improve the symptoms of AD. To move the field forward and accomplish this unmet need, a deeper understanding of the underlying disease mechanisms is needed, leading to new therapeutic targets that then can be used in drug development, and therefore, academia, biopharmaceutical companies, investors, government, and the patients must work together.

Several important conceptual shifts around AD have advanced the field significantly, such as moving from the syndrome, based on signs and symptoms, to a biomarker diagnosis based on the pathological hallmarks of the disease: amyloid β (Aβ) deposition, pathologic tau, and neurodegeneration [2]. Additionally, genetic risk factors for the sporadic non-familial form of AD are emerging, providing further insight into the molecular underpinnings of the disease [2]. Both these shifts and insights resulted in more target classes to investigate for AD drug development.

However, over the past two decades, AD drug development has been particularly challenging. The failure of most AD trials to show efficacy has prevented new treatments from reaching the market, with the exception of the approval of Biogen’s aducanumab by the US Food and Drug Administration (FDA). The reasons for these failures are manyfold and have been discussed elsewhere [3]. However, despite these failures in the past, the field has not lost confidence in drug development for AD, as novel drug development strategies are emerging.

In this review, we aim to provide a unique overview of the drug development landscape for AD. In particular, by comparing drugs in preclinical development by biotechnology or pharmaceutical companies to drugs currently tested in clinical trials, we identify trends regarding target classes and treatment modalities used and discuss innovations in AD drug development that hopefully eventually will be tested in clinical trials.

## Methods

### Selection of drugs

#### Drugs in preclinical development

We included drugs that are in development by biotechnology or pharmaceutical companies to provide a dataset of the most relevant drugs selected by the industry for development. To that end, we analyzed proprietary and public databases for drugs in preclinical development for AD. All drugs were manually validated by a rater to have a valid source, such as a pipeline on a company website, a press release, or a publication, within the last 5 years (2016–2020). The 5-year time window was set to limit the inclusion of programs that are not currently active. If the only source was a company website, it was left to the interpretation of the rater whether the website was up to date, taking the 5-year time window into account. Patents were not included as a source. If an intervention was in clinical development for a different indication but in preclinical development for AD, the intervention was classified as preclinical. If a drug was renamed, the drug name was adapted and scored accordingly. All drugs analyzed were labeled by the rater as “verified,” “unverifiable,” or “to be discussed.” Drugs labeled as “to be discussed” were further assessed for validity and appropriate scoring in consensus meetings among six raters.

#### Drugs in clinical development

The clinical trial registries from the USA (ClinicalTrials.gov), Europe (eudract.ema.europa.eu), Asia (chictr.org.cn, cris.nih.go.kr, and umin.ac.jp/ctr), and Australia (anzctr.org.au) were assessed in December 2020 for clinical trials with “Alzheimer’s disease” as the indication. Although some trials in other countries might have been missed, most trials in the four major continents where AD trials take place will be identified, when appropriately registered. Generally, compliance with the required trial registration is considered to be high among trial sponsors [4]. All trials of all drugs in phases 1, 2, and 3 were included; phase 1/2 and phase 2/3 studies are listed as phase 1 and phase 2 studies, respectively. We included trials that are recruiting, not yet recruiting, active, not recruiting, and enrolling by invitation. We did not include trials listed as completed, suspended, unknown, withdrawn, or terminated. We did not include trials of non-pharmacologic therapeutic approaches such as cognitive therapies, caregiver interventions, supplements, health tech interventions, and medical foods. We did not include trials of biomarkers. If there were multiple trials ongoing with an intervention, we included only the trial that was most advanced in development for AD. If an intervention was in multiple stages of clinical development for AD, we listed only the most advanced stage. Trial extensions (e.g., open-label or closed label extensions of a completed double-blind phase of a trial) were defined as “already listed,” and only the original trial was scored and included in the analysis. Interventions listed in multiple trial registries were included only once.

### Scoring of drugs

#### Target class

For all drugs, the primary drug target was identified and classified according to the terminology of the Common Alzheimer’s and Related Dementias Research Ontology (CADRO) [5, 6]. CADRO systematizes the pathological underpinnings of AD that are the current drug targets relevant to AD and provides a framework for classifying treatment mechanisms. The following target classes for AD are defined in CADRO: amyloid, tau, apolipoprotein E (ApoE)/lipids/lipoprotein receptors, neurotransmitter receptors, neurogenesis, inflammation, oxidative stress, cell death, proteostasis/proteinopathies, metabolism/bioenergetics, vasculature, growth factors/hormones, synaptic plasticity/neuroprotection, epigenetics, gut-brain axis, circadian rhythm, environmental factors, others, and unknown. As some drugs did not fall within any of these classes, the CARDO target classes were complemented with the following classes: ER stress/cellular stress, lysosomal/endosomal and autophagy, and antiviral/antibacterial. Target classes are defined based on the primary target of the active ingredient, not on any possible downstream effects. If there were multiple mechanisms of action, a literature search was performed to establish the most dominant mechanism or the target class and the agent was classified as “multitarget.”

#### Treatment modality

Every drug was classified by the chemical structure of the active ingredient [7]. Drugs were classified into the following groups: small molecule, antibody, enzyme/protein, peptide, DNA, RNA, natural product, drug combination, cell therapy, aptamer, bacteria/probiotic, antibody combination, inorganic, others, and unknown.

For all drugs, the drug target and the sponsor (either the company name, funding agency, or non-profit organization) were noted. For all drugs in clinical development, we noted the trial registry or registries in which the drug is listed, the development stage, and the year of study start.

## Results

By the end of 2020, a total of 441 drugs in development for the treatment of AD were selected as eligible for scoring, of which 291 (66%) were in the preclinical stage of development, and 150 drugs (34%) were in clinical stages of development (Additional file 1, number of drugs in preclinical and clinical development for AD.xls). These 441 drugs are categorized into 22 target classes and 13 treatment modalities. Most drugs were aimed at Aβ (*n* = 72), inflammation (*n* = 56), neurotransmitters and receptors (*n* = 49), and tau (*n* = 44).

### Target classes: preclinical vs clinical

**Fig. 1:**
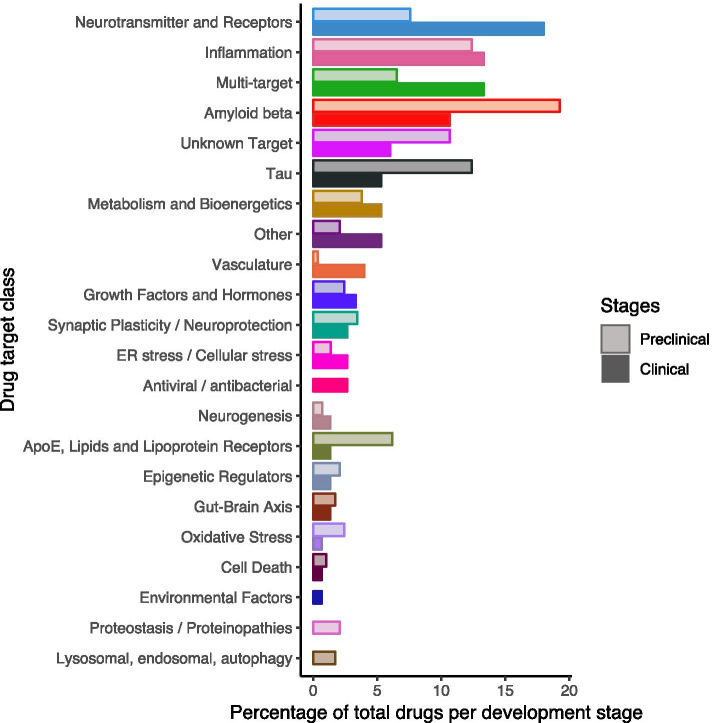
AD drugs in development, preclinical vs clinical. The number of drugs per target class in preclinical development is shown as a percentage of all drugs in preclinical development, and the number of drugs per target class in clinical development is shown as a percentage of all drugs in clinical development

To allow for comparison of the relative number of drugs per target class in preclinical development vs clinical development, Fig. 1 shows the percentage of drugs per target class in preclinical development among all drugs in preclinical development and the percentage of drugs per target class in clinical development among all drugs in clinical development. We observed a large difference between clinical (18%) and preclinical (8%) drugs targeting neurotransmitters and receptors. This class includes for example cholinesterase inhibitors and a partial NMDA receptor antagonist, which were among the first approved drugs for symptomatic treatment for AD.

Relatively more drugs targeting Aβ were found in preclinical development (19%) as compared to clinical development (11%). As amyloid is the earliest and best described pathological hallmark of AD, over the last two decades, much effort has focused on drug development targeting amyloid. However, although several treatments were able to clear Aβ deposits from the brains of AD patients, cognitive effects were often ambiguous [8]. Therefore, the need to diversify the AD pipeline in terms of targets has been championed [9]. While we observed that the drug development strategy is being diversified, our data indicate that amyloid-targeting therapies remain a prominent avenue of development.

Tau, the second pathological hallmark of AD, became more popular as a drug target a bit later than amyloid, especially when it became evident that tau accumulation is directly associated with cognitive decline in AD, where Aβ accumulation is suggested to have an indirect, tau-mediated association with neurodegeneration and clinical manifestations of AD [10]. Tau is an increasingly popular target as demonstrated by the fact that only 5% of the total drugs in clinical development (8 drugs) target tau, vs 12% of total drugs in preclinical development (36 drugs).

Targeting pathways involved in (neuro)inflammation remains a strategy of interest both in preclinical and clinical development [11]. Inflammation is recognized as a promising target for treatment development in AD. It is the second most popular class in both clinical development (13%, 20 drugs) and preclinical development (together with “tau,” also 12%, 36 drugs).

When considering other targets, a substantial increase is observed for novel target classes in preclinical development. These include ApoE/lipids/lipoprotein receptors, proteostasis/proteinopathies, lysosomal/endosomal targets, and autophagy. These are all biological processes tightly linked to the genetic underpinnings of sporadic AD [12].

### Drugs in development per clinical phase

**Fig. 2:**
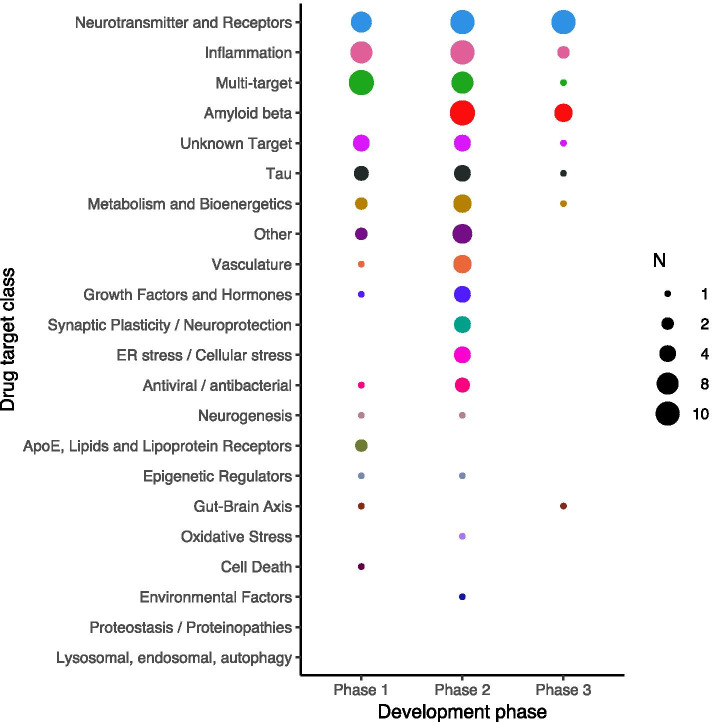
Number of AD drugs by clinical development stage, stratified by target class. The size of the dotted circles indicates the number of drugs as reflected in the legend

In a standard drug development pyramid, the number of drugs decreases from phase 1 to phase 3, because of safety issues or lack of efficacy. However, in AD, more drugs are in phase 2 of development compared to phase 1 (Fig. 2). This is likely in part because of the use of repurposed compounds that enter at phase 2 without phase 1 and because phase 1 studies are only several weeks in duration while in AD phase 2 studies tend to be much longer in order to be able to show a treatment effect on a biomarker or on cognitive decline [13]. Furthermore, we found that few interventions go from phase 2 to 3, in particular for target classes that are not directly linked to pathology or genetic risk factors, such as metabolism and bioenergetics, vasculature, and growth factors and hormones. This could be caused by the lack of biomarkers to show (hints of) efficacy for these types of drugs. For a detailed breakdown of drugs in clinical development for AD, we refer to Cummings et al. [14].

### Treatment modalities of AD drugs

**Fig. 3:**
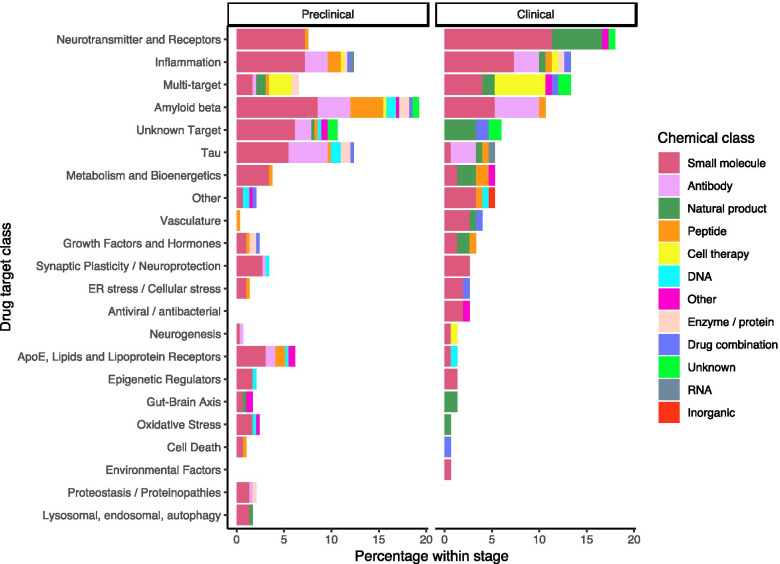
AD drugs per target class, stratified by treatment modality

The chemical structure of the active ingredient determined the class of treatment modality of the scored drugs. More novel treatment modalities, such as enzyme/protein therapy and gene therapy (DNA/RNA), are mostly in preclinical stages, while small molecules are in all stages of development (Fig. 3). Interestingly, novel treatment modalities are usually developed on more traditional targets such as amyloid and tau, while on new targets, mostly traditional modalities such as small molecules are developed. For amyloid therapies in clinical development, mostly antibodies are used, while in the preclinical stage of development, amyloid is targeted mostly using small molecules. Of note, about half of the multitarget interventions are cell therapies.

## Discussion

By reviewing AD drugs currently in preclinical development, we provide insight into the future clinical development pipeline. Moreover, comparing drugs currently in preclinical development to drugs in clinical development provides knowledge into how the landscape is evolving.

### Gaps and trends

We observed that amyloid-targeting therapies are increasingly being developed in the preclinical setting. This is surprising as several treatments in clinical trials over recent years were able to clear Aβ deposits from the brains of AD patients while cognitive effects were often ambiguous [8]. Strategies entailed for example reduction of Aβ peptide production (such as β-site amyloid precursor protein cleaving enzyme (BACE) inhibitors and γ-secretase inhibitors) or clearance of Aβ peptides and Aβ protein aggregates (such as Aβ-directed antibodies). When treating early symptomatic or asymptomatic patients with BACE inhibitors, cognition worsened [15]. Moreover, funding agencies such as the Alzheimer’s Disease Discovery Foundation, as well as investors such as the Dementia Discovery Fund, have put policies in place to promote diversification of AD interventions and therefore diminution of amyloid targeting drugs. A possible explanation for our data is that many of the amyloid-targeting drugs in the clinic are monoclonal antibodies, and funders are searching for small molecules with a similar plaque-reducing effect that are much less costly to produce and easier to administer. The recent FDA approval of aducanumab might be followed by several more amyloid-directed therapies.

Apart from pathological hallmarks used as drug targets for AD, the genetic architecture of the late-onset AD (LOAD) is increasingly being elucidated [16], providing additional avenues for drug development. About 30 common susceptibility loci are known to significantly affect disease risk and rare variants also contribute to disease risk [17–19]. A substantial number of these risk genes are expressed on microglia, myeloid cells in the brain that are involved in the regulation of the immune response, inflammation, and clearance of protein aggregates and lipoproteins from the interstitial fluid. These include, for example, the lipoprotein receptor triggering receptor expressed on myeloid cells 2 (TREM2), which may influence neurodegeneration, possibly through clearance of lipoprotein aggregates; CD33 which may play a role in protein clearance and other neuroinflammatory pathways mediated by microglia; and phospholipase C gamma 2 (PLCG2) regulating inflammatory and metabolic responses in microglia [20]. These and other risk factors have substantiated the significant role of neuroinflammation and dysregulation of the immune response play in AD pathology. This is also reflected in our data of preclinical and clinical drugs in development targeting inflammatory pathways.

The strongest genetic risk gene for AD remains APOE. This was the first risk gene identified for LOAD [21], and APOE genotype affects the risk of familial and early forms of AD as well. Risk is dose-dependent, with the major risk variant APOE4 contributing a threefold increase in AD among E4 heterozygotes and a 15-fold increase in AD among E4 homozygotes. ApoE protein acts as the major lipoprotein carrier in the brain. In addition to APOE, several other LOAD risk genes, such as ATP-binding cassette A7 (ABCA7), TREM2, clusterin (CLU), and sortilin 1 (SORL1), are involved in lipid metabolism. ABCA7 influences AD risk via a reduced ability to transfer cholesterol to ApoE, TREM2 variants affect the uptake of lipoproteins on microglia cells, CLU (apolipoprotein J) plays a role in lipid transport, and SORL1 acts as an ApoE receptor on neuronal cells [22]. Considering the major role these functional genomic pathways involving lipid transport and metabolism play in the pathogenesis of AD [23], the observation that only 2 drugs (1%) in clinical development target these pathways can be considered a major gap in the AD drug development pipeline. This trend might change because in preclinical development, lipid metabolism is increasingly targeted (18 drugs, 6%).

A similar observation was made for drugs targeting endosomal and lysosomal pathways, with 5 drugs (2%) in preclinical development and none in clinical development. This can be considered another gap in the pipeline as multiple risk genes point towards these pathways for AD drug development. For example, phosphatidylinositol-binding clathrin assembly protein (PICALM)-mediated Aβ generation and clearance may influence the accumulation of Aβ fragments in AD brains [24], bridging integrator 1 (BIN1) internalizes Aβ peptides and ApoE via the endosomal-lysosomal pathway [25], and SORL1 directs APP to the endocytic pathways for recycling [22].

### Limitations

In order to identify drugs in preclinical development for AD, we set out to identify therapeutics that are developed by biotechnology or pharmaceutical companies. The goal here is not to provide the most comprehensive list of all therapeutics in preclinical development for AD, but instead to provide a dataset of the drugs most relevant to the industry for development. It should be noted that some drugs in preclinical development, especially those funded by governmental agencies are under-represented in our review; early drug development for the industry is increasingly performed in academic laboratories [26].

In our selection of drugs in clinical development stages, all clinical trials were included regardless of the sponsor of the study, whereas the preclinical stage drugs were limited to treatments in development and financed at biotech or pharmaceutical companies. Differences between these stages might therefore relate not only to chronologic development trends over time, but may also reflect differences in (strategic) interests from companies vs non-industry investors. For example, financially less attractive treatment options where limited return on investments is expected, such as repurposed drugs, might be pursued by academia and can therefore be more highly represented in clinical stages. In our dataset, drugs in clinical development include 71 drugs with an academic or non-industry sponsor. However, when we exclude these 71 drugs from the analysis (data not shown), trends and conclusions remain unchanged.

## Conclusions

From our data, we conclude that in drug development for AD, amyloid-targeting therapies are increasingly being developed in the preclinical setting, neuroinflammatory targets are prominent in both preclinical and clinical development, and preclinical AD drugs are increasingly targeting the molecular pathways associated with sporadic AD genetic risk variants. These observations have important implications for how the AD drug development landscape will involve in the coming years.

### Implications for AD drug development

Once the preclinical therapies reach the clinic, the field will need to be prepared for the assessment of their efficacy in patients. Biomarkers will need to be available that can show pharmacodynamic effects and early signs of efficacy [3]. This will be essential for the selection of drugs suitable to progress from early clinical development to phase 3 trials. This requires substantial investment in the development of biomarkers. Examples include the development of fluid biomarkers to measure lipoprotein levels and lipid metabolism in cerebrospinal fluid [27], and markers of endosomal/lysosomal activity. These markers will require clinical validation in AD cohorts and memory clinics. Also, brain imaging modalities such as positron emission tomography (PET) tracers quantifying inflammation, lipid metabolism and buildup, and endosomal/lysosomal capacity will need to be developed. Furthermore, genetic screening of patients for sporadic AD genetic risk variants will need implementation to allow for stratification of patients to be included in trials, to reduce the heterogeneity of patients and to allow for more personalized therapy development directed at the etiology of the disease in subsets of patients.

### Future perspectives

Drug development for AD is and will remain a challenge. The complexity of the disease with respect to the genetic risk factors, the pathological underpinnings, and the progressive nature requires a multitude of drug development strategies. The onset of pathological processes far ahead of the initial symptoms further complicates the intervention strategies as this requires early detection and therapeutic intervention [28]. Despite these challenges, substantial progress has been made in recent years. Drug development shifted from symptomatic interventions to drugs targeting AD pathology. On June 7, 2021, this culminated in the first FDA approval of a disease-modifying drug for AD, the fully human anti-amyloid monoclonal antibody aducanumab. Despite the controversy around the approval, this is a major step for the field. Discussing the clinical meaningfulness of the two phase 3 trials of aducanumab goes beyond the scope of this paper. However, we are hopeful a drug approval by the FDA will be reflected in an increase in the number of preclinical and clinical intervention studies in AD. As such, our data reported here may well serve as a baseline for a comparison in the coming years.

The field and AD patients, in particular, will require drugs that are effective and can halt progression or even prevent the disease. Drugs targeting functional genetic pathways leading to AD pathology are arguably best suited for preventive strategies and early intervention. Drugs targeting genetic pathways of neuroinflammation are currently in clinical stages, and we found the emergence of the first treatments in preclinical development targeting the lipid metabolism and endosomal/lysosomal pathways. When the AD drug development field continues to invest in the diversification of drug targets and diagnostics, we are hopeful for the next generation of AD drugs that will soon enter clinical trials.

## Supplementary Information

**Additional file 1 **: **Table S1.** Number of drugs in preclinical (pre) and clinical (clin) development for AD, stratified by target class and treatment modality.

## Data Availability

The datasets analyzed during the current study are not publicly available due to the proprietary nature of some of the databases used, but the information is available from the corresponding author on reasonable request with the permission of third parties. Data resulting from drug scoring are available in the additional data files.

## Abbreviations

AD: Alzheimer’s disease
FDA: Food and Drug Administration
ApoE: Apolipoprotein E
Aβ: Amyloid beta
BACE: β-Site amyloid precursor protein cleaving enzyme
LOAD: Late-onset AD
TREM2: Triggering receptor expressed on myeloid cells 2
PLCG2: Phospholipase C gamma 2
ABCA7: ATP-binding cassette A7
CLU: Clusterin
SORL1: Sortilin 1
PICALM: Phosphatidylinositol-binding clathrin assembly protein
BIN1: Bridging integrator 1
PET: Positron emission tomography
